# Sewage in Scotch Lochs

**Published:** 1895-10-26

**Authors:** 


### Sewage in Scotch Lochs.

It is a prevailing delusion that sewage, whilst very destructive in fresh water, is rendered comparatively harmless so soon as it comes in contact with the salt water of the sea. Science could not, of course, encourage any such doctrine. Sea-water can no doubt render innoxious a strictly limited quantity of sewage; but when large quantities are poured into estuaries and bays whose shores are peopled by considerable populations, there are many dangers to health of which little account is taken. Dr. McVail, medical officer of health for the County Council of Dumbarton, gives, in his annual report for 1895, some very striking facts in this connection. If any animals can stand sewage it would be, one would think, the comparatively low forms of life which are found between the tide-marks on our British shores. They must have had as considerable a training in digesting filth as any creatures in the world. Nevertheless, in the Gareloch and other small sea lochs of the Firth of Clyde it is found that certain very robust molluscs, more particularly *Mya arenaria* and *Mya truncata*, which used to be very abundant in these waters, are now almost extinct, having been poisoned by the filthy outpourings of Glasgow and other towns in the neighbourhood. That this wholesale destruction of animal life has been produced by sewage and nothing else is proved by the fact that at particular portions of the lochs, comparatively little touched by the sewage, these very same molluscs still thrive as abundantly as in happier times. On the other hand, sewage itself has a charm for certain varieties of crustaceans; and these thrive mightily in the vile water which has destroyed the molluscs. Persons who are fond of crabs, and especially of certain favourite portions of them, will do well to remember that the crab is a typical crustacean, as is also the still more favourite lobster. There are a good many morals which men and women of a little intelligence may draw from these facts. One is that the shores of the Clyde lochs are not very nice places, more particularly for visitors in hot summer weather; and another, that molluscs, and crabs and lobsters also, which are bred and fed in such localities can hardly be as wholesome eating as a fastidious person could desire.

